# Flavonoid-converting capabilities of *Clostridium butyricum*

**DOI:** 10.1007/s00253-025-13434-0

**Published:** 2025-02-27

**Authors:** Annett Braune

**Affiliations:** Research Group Intestinal Microbiology, Department of Molecular Toxicology, German Institute of Human Nutrition Potsdam-Rehbruecke, Nuthetal, Germany

**Keywords:** *Clostridium butyricum*, Flavonoid, Flavonoid glycoside, Polyphenol, Gut bacterium, Soil bacterium

## Abstract

**Abstract:**

*Clostridium butyricum* inhabits various anoxic environments, including soil and the human gut. Here, this common bacterium comes into contact with abundant plant-derived flavonoids. Metabolization of these bioactive polyphenols has been studied in recent years, particularly focusing on gut bacteria due to the proposed health-promoting properties of these dietary constituents. Based on an initial report in 1997 on eriodictyol degradation (Miyake et al. 1997, J Agric Food Chem, 45:3738–3742), the present study systematically investigated *C. butyricum* for its ability to convert a set of structurally diverse flavonoids. Incubation experiments revealed that *C. butyricum* deglycosylated flavonoid *O*-glucosides but only when glucose was absent. Moreover, aglycone members of flavone, flavanone, dihydrochalcone, and flavanonol subclasses were degraded. The C-ring cleavage of the flavanones, naringenin and eriodictyol, was stereospecific and finally resulted in formation of the corresponding hydroxyphenylpropionic acids. Stereospecific C-ring cleavage of the flavanonol taxifolin led to taxifolin dihydrochalcone. *C. butyricum* did neither cleave flavonols and isoflavones nor catalyze de-rhamnosylation, demethylation, or dehydroxylation of flavonoids. Genes encoding potential flavonoid-metabolizing enzymes were detected in the *C. butyricum* genome. Overall, these findings indicate that *C. butyricum* utilizes flavonoids as alternative substrates and, as observed for the dihydrochalcone phloretin, can eliminate growth-inhibiting flavonoids through degradation.

**Key points:**

*• Clostridium butyricum deglycosylated flavonoid O-glucosides.*

*• Clostridium butyricum converted members of several flavonoid subclasses.*

*• Potential flavonoid-metabolizing enzymes are encoded in the C. butyricum genome.*

## Introduction

**Fig. 1:**
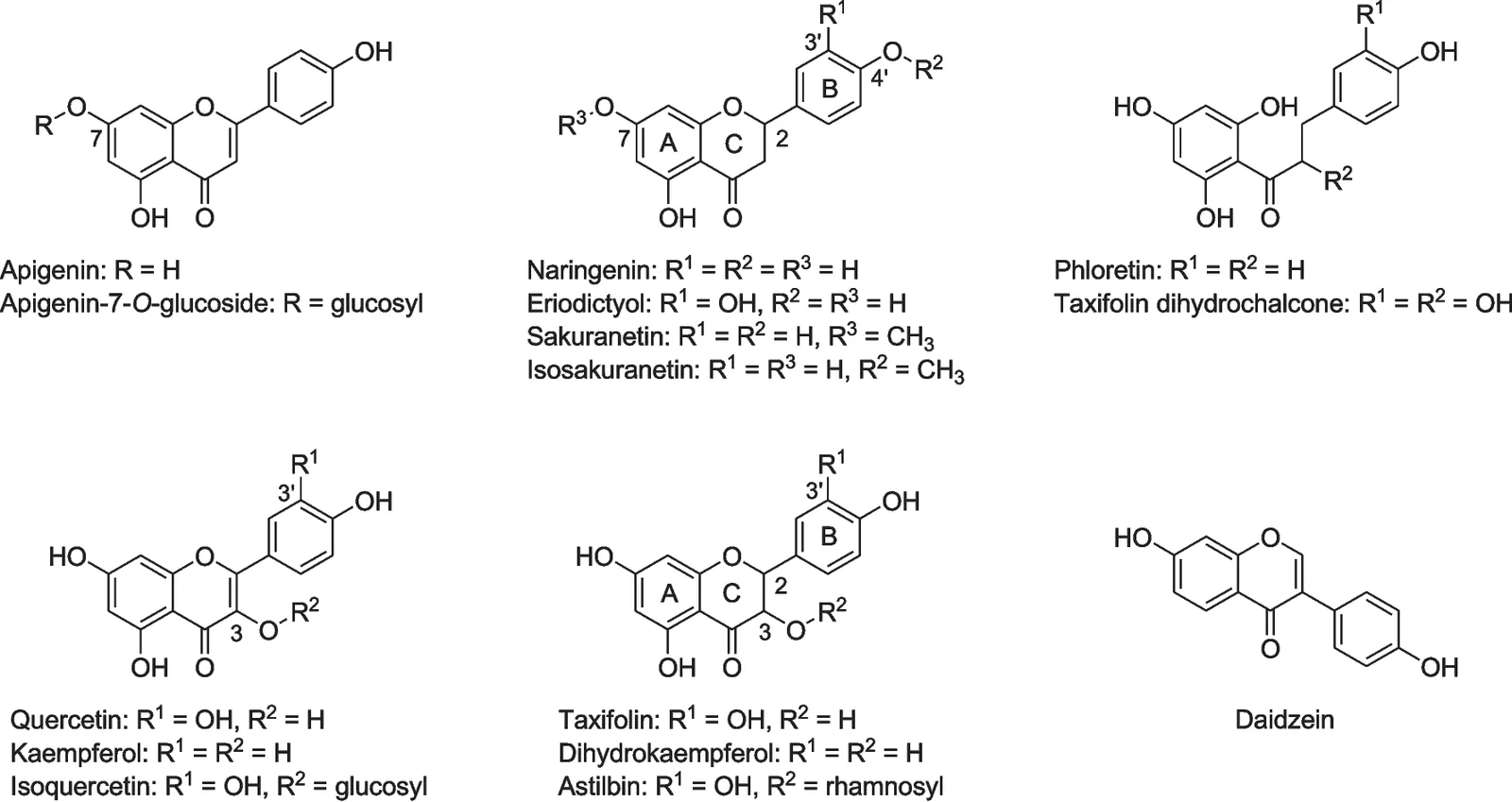
Structures of flavonoids used in the study

Flavonoids are a prevalent group of polyphenols synthesized by plants and, consequently, abundant in soil through litter decomposition. As constituents of plant-derived foods and beverages, flavonoids are consumed by humans (Williamson 2017). An inverse association between flavonoid intake and the risk of various chronic diseases, such as cardiometabolic disorders, has been reported and is backed up by a wide range of biological effects demonstrated in experimental studies (Fraga et al. 2019; Oteiza et al. 2018; Rodriguez-Mateos et al. 2024; Williamson et al. 2018). Following their consumption, most flavonoids are poorly absorbed and metabolized by the human body. Thus, these compounds may enter the large intestine becoming available for metabolization by gut microbiota. As the beneficial health effects of consumed flavonoids may actually be mediated by their metabolites (Espín et al. 2017; Fraga et al. 2019; Osborn et al. 2021; Williamson et al. 2018), the microbial contribution to flavonoid conversion needs to be clarified. As most flavonoids are present mainly in glycosidic form (for structures, see Fig. 1), deglycosylation is the first step of their conversion and required prior to further degradation. The deglycosylation of flavonoids is carried out by a substantial number of gut bacterial species, whereas conversion of the resulting aglycones is a rare trait within the human gut microbiota (Braune and Blaut 2016; Goris and Braune 2024). The metabolism of flavonoids by strictly anaerobic bacteria in the gut proceeds under conditions comparable to those in anoxic soils; but the latter subject has been less studied (McGivern et al. 2021).

*Clostridium butyricum* inhabits various anoxic ecosystems, including the mammalian gut and the soil. The first strain has been isolated from pig intestine in 1880 by Adam Prażmowski at the University of Leipzig, who subsequently assigned its binomial name *Clostridium butyricum* (Prazmowski 1880; Skerman et al. 1980) as the first of the *Clostridium* genus. *C. butyricum* is a common bacterium of human and animal gut microbiota, although certain strains have been implicated in pathological conditions (Cassir et al. 2016). The species has been detected method-dependently in feces of 20 up to 100% of adults, but its abundance appeared to be very low (0.004%) (Candeliere et al. 2023; Finegold et al. 1983). Regarding food and environmental samples, *C. butyricum* strains have been found mainly in soil, vegetables, soured milk, and cheeses (Ghoddusi and Sherburn 2010). For decades, *C. butyricum* has been used as a probiotic in Asia (Stoeva et al. 2021) and is now also approved in Europe as both feed additive and food ingredient. The observed therapeutic and potentially protective effects of *C. butyricum* in various diseases are currently investigated in order to clarify the underlying molecular mechanisms. In this context, generation of short-chain fatty acids (SCFA), in particular butyrate, from dietary fibers has been acknowledged as the most obvious feature of *C. butyricum* but other mechanisms to promote host health are being explored (Pickens and Cockburn 2024; Stoeva et al. 2021).

In an early study, *C. butyricum* has been reported to degrade the flavonoid eriodictyol (structure in Fig. 1) present in lime fruit to 3-(3,4-dihydroxyphenyl)propionic acid and phloroglucinol (Miyake et al. 1997). This would implicate cleavage of the central C-ring of the flavanone structure but has not been followed up to date. The few human gut bacteria so far known to catalyze this reaction are members of *Eubacteriaceae* (*Eubacterium ramulus*), *Oscillospiraceae* (*Flavonifractor plautii*), or *Lachnospiraceae* (*Catenibacillus scindens* and *Catenibacillus decagia*), all belonging to the phylum *Bacillota* (Braune and Blaut 2018, 2011; Braune et al. 2001; Goris and Braune 2024; Schneider and Blaut 2000; Schoefer et al. 2003; Winter et al. 1991). Thus, *C. butyricum* would represent the first human intestinal *Clostridiaceae* species capable of flavonoid conversion.

The present study aimed at evaluating the ability of *C. butyricum* to metabolize a tailored selection of flavonoids representing several subclasses and substitution patterns. Incubation experiments were performed under anoxic conditions to test for *O*-deglycosylation, demethylation, or dehydroxylation of applied flavonoids and to elucidate the cleavage of the basic flavonoid structure. Based on the catalyzed reactions and literature data, the genome of *C. butyricum* was examined for encoded enzymes likely to being involved in flavonoid conversion by this bacterium.

## Materials and methods

### Chemicals

Apigenin, apigenin-7-*O*-glucoside, eriodictyol, isoquercetin (quercetin-3-*O*-glucoside), naringenin, phloretin, quercetin, and ( +)-taxifolin were purchased from Roth (Karlsruhe, Germany). Eriodictyol and naringenin were identified previously as racemic compounds by chiral HPLC analysis (Braune et al. 2019). ( ±)-Taxifolin, ( ±)-trans-dihydrokaempferol, 3-(4-hydroxyphenyl)propionic acid, 3-(3,4-dihydroxyphenyl)propionic acid, and phloroglucinol were obtained from Sigma-Aldrich (Munich, Germany). Astilbin (taxifolin-3-*O*-rhamnoside) and kaempferol were purchased from abcr (Karlsruhe, Germany), sakuranetin (7-*O*-methyl-naringenin), and isosakuranetin (4′-*O*-methyl-naringenin) from Extrasynthese (Genay Cedex, France), and daidzein from Acros Organics (Geel, Belgium). Taxifolin dihydrochalcone (2-hydroxy-1-(2,4,6-trihydroxyphenyl)-3-(3,4-dihydroxyphenyl)propan-1-one) was available from a previous study (Braune et al. 2019).

### Bacterial strain and cultivation

*Clostridium butyricum* DSM 10702^ T^ (German Collection of Microorganisms and Cell Cultures, DSMZ; Braunschweig, Germany) was routinely grown under strictly anoxic conditions in 16-mL Hungate tubes containing 10 mL of medium with a gas phase of N_2_/CO_2_ (80:20, v/v) at 37 °C for 14 h. A modified reinforced clostridial medium without agar and starch (RCM_mod_) was applied, which contained (g/L): yeast extract (3.0), meat extract (10), peptone (10), glucose (5.0), NaCl (5.0), sodium acetate (3.0), and cysteine hydrochloride (0.5). Optical density at 600 nm (OD_600_) was measured using a spectrophotometer (SmartSpec Plus; Bio-Rad, Feldkirchen, Germany).

### Flavonoid conversion tests

For incubation experiments, RCM_mod_ containing glucose as described above or RCM_mod_ lacking glucose was used. The medium (940 µL) was supplemented with 10 µL of a 20 mM flavonoid stock solution prepared in dimethyl sulfoxide (DMSO) (final concentration: 200 µM) and inoculated with 50 µL of an overnight culture of *C. butyricum.* The test cultures were incubated up to 144 h at 37 °C in an anoxic workstation (MACS Anaerobic Workstation, Don Whitley Scientific, Shipley, UK) containing a gas atmosphere of N_2_/CO_2_/H_2_ (80:10:10, v/v/v). Samples (150 µL) were withdrawn at the times indicated.

For studies on antibacterial effects, 16-mL Hungate tubes containing 5 mL of RCM_mod_ with glucose and supplemented with 53 µL of a 20 mM stock solution of phloretin or naringenin in DMSO (final concentration: 200 µM) were used. Media were inoculated with 250 µL of an overnight culture of *C. butyricum* and incubated at 37 °C. Samples (200 µL) were taken with a syringe at the times indicated over a period of 144 h.

Flavonoids and bacteria incubated separately in medium served as controls. The incubations were carried out in duplicate or triplicate.

### HPLC analysis

Samples from conversion tests were processed and analyzed by reversed-phase high-performance liquid chromatography–diode array detection (RP-HPLC/DAD) using a LiChrospher 100 RP-18 column as described recently (Goris and Braune 2024). To confirm the identity of taxifolin dihydrochalcone, an additional HPLC method using a Zorbax SB-C_18_ column (Braune et al. 2019) was applied.

For chiral analyses at the same HPLC system as above, a Daicel Chiralpak AD-RH column (150 × 4.6 mm, 5 µm; Chiral Technologies, Illkirch, France) equipped with a corresponding guard column was used. The column temperature was kept at 30 °C. The mobile phase was a mixture of 0.1% (v/v) aqueous trifluoroacetic acid (solvent A) and methanol (solvent B). For analysis of samples from naringenin conversion, solvents were supplied in a gradient mode (B from 30 to 90% in 10 min, held for 40 min) at a flow rate of 0.4 mL/min. Samples of conversion experiments with eriodictyol and taxifolin were analyzed using individual gradients (eriodictyol: B from 30 to 90% in 20 min, held for 20 min; taxifolin: B 30% for 10 min, 40% for 20 min, from 40 to 90% in 10 min, and from 90 to 100% in 20 min) at a flow rate of 0.8 mL/min.

Calibration curves of the corresponding standard compounds were used for quantification, except of taxifolin dihydrochalcone, which was quantified based on the taxifolin standard.

### Sequence analyses

Sequence similarity searches and sequence comparison were performed with the Basic Local Alignment Search Tool (BLAST, https://blast.ncbi.nlm.nih.gov/Blast.cgi). Amino acid sequence identity of at least 30% for pairwise comparison of complete sequences was considered. Searching for putative transcription promoter and terminator sequences was done using the web-based tools BPROM (www.softberry.com) and ARNOLD (http://rna.igmors.u-psud.fr/toolbox/arnold), respectively. Signal peptide likelihood on the N-terminus of protein sequences was determined by the web tool SignalP-6.0 (https://dtu.biolib.com/SignalP-6) and the expected sub-cellular location of proteins by ProtCompB (www.softberry.com). An analysis of protein sequences for conserved domains was done on the NCBI website (https://www.ncbi.nlm.nih.gov/Structure/cdd/wrpsb.cgi).

## Results

### Flavonoid conversion by *C. butyricum*

**Fig. 2:**
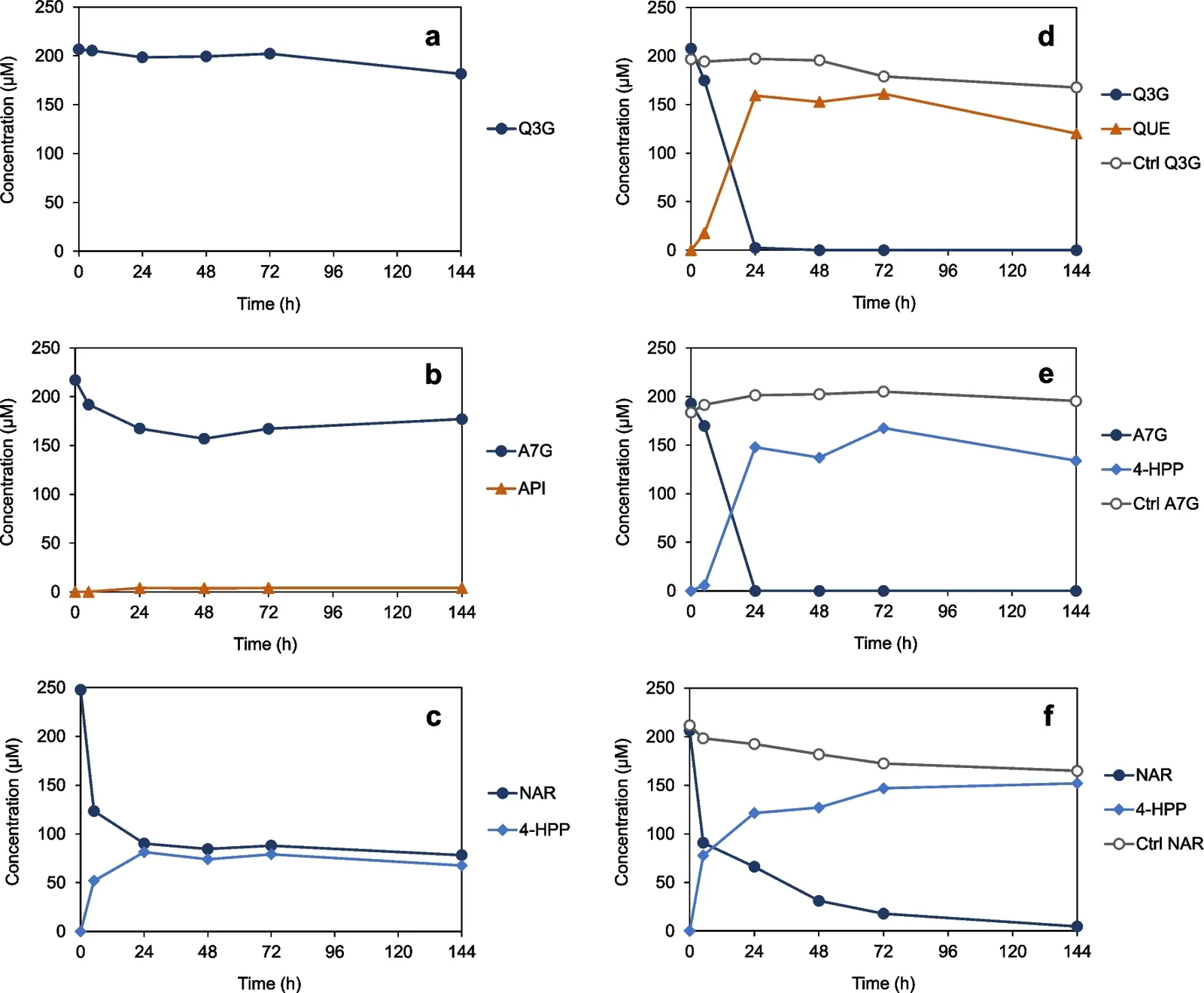
Deglycosylation of flavonoids by *C. butyricum* was inhibited by glucose present in medium. **a, b**, and** c** Incubation of flavonoids with *C. butyricum* in RCM_mod_ with glucose: **a** isoquercetin (quercetin-3-*O*-glucoside, Q3G), **b** apigenin-7-*O*-glucoside (A7G), and **c** naringenin (NAR). **d, e**, and** f** Incubation of flavonoids with *C. butyricum* in RCM_mod_ without glucose: **d** Q3G, **e** A7G, and **f** NAR. As controls (Ctrl), flavonoids were incubated in RCM_mod_ without glucose in the absence of *C. butyricum*. Data points are means of duplicates. QUE, quercetin; API, apigenin; 4-HPP, 3-(4-hydroxyphenyl)propionic acid

Initial conversion tests with *C. butyricum* in the routinely applied glucose-containing RCM_mod_ compared to RCM_mod_ lacking glucose revealed the deglycosylation of flavonoids (200 µM) to be influenced by the presence or absence of this monosaccharide. When glucose was present, isoquercetin (quercetin-3-*O*-glucoside) was not converted to any product within 144 h of incubation (Fig. 2a), and from apigenin-7-*O*-glucoside, only very small amounts of apigenin were formed (Fig. 2b). In glucose-free medium, isoquercetin (Fig. 2d) and apigenin-7-*O*-glucoside (Fig. 2e) were completely converted within 24 h of incubation to quercetin and 3-(4-hydroxyphenyl)propionic acid (4-HPP), respectively. This glucose-dependent behavior could not be explained by differences in growth of the glucose-fermenting *C. butyricum*. Grown in RCM_mod_ with glucose, *C. butyricum* reached a maximum OD_600_ of 7.2 after 24 h of incubation, while its cultivation without glucose led to an OD_600_ of only 0.5 after the same time. The initial conversion of a selected flavonoid aglycone, naringenin, did not considerably differ depending on glucose content in medium. Within 6 h of incubation, naringenin was converted at similar rates to 4-HPP in the presence or absence of glucose (Fig. 2c and f). However, differences were observed at later time points of incubation due to the presence of two naringenin enantiomers, as discussed below.

Based on the results of these initial experiments with the two media, subsequent investigations on conversion of flavonoids (200 µM) by *C. butyricum* were conducted in RCM_mod_ without glucose. Flavonoid glycosides and aglycones representing several flavonoid subclasses and comprising varying substitution patterns were included (structures in Fig. 1). Flavonoid subclasses and individual members were primarily selected according to their presence in foods and associated biological effects reported previously (Fraga et al. 2019; Manach et al. 2004). However, several of these flavonoids are also released into the soil by root exudation or plant tissue degradation, where they play important roles in the rhizosphere, such as mediating plant–microbe interactions (Wang et al. 2022; Weston and Mathesius 2013).

**Fig. 3:**
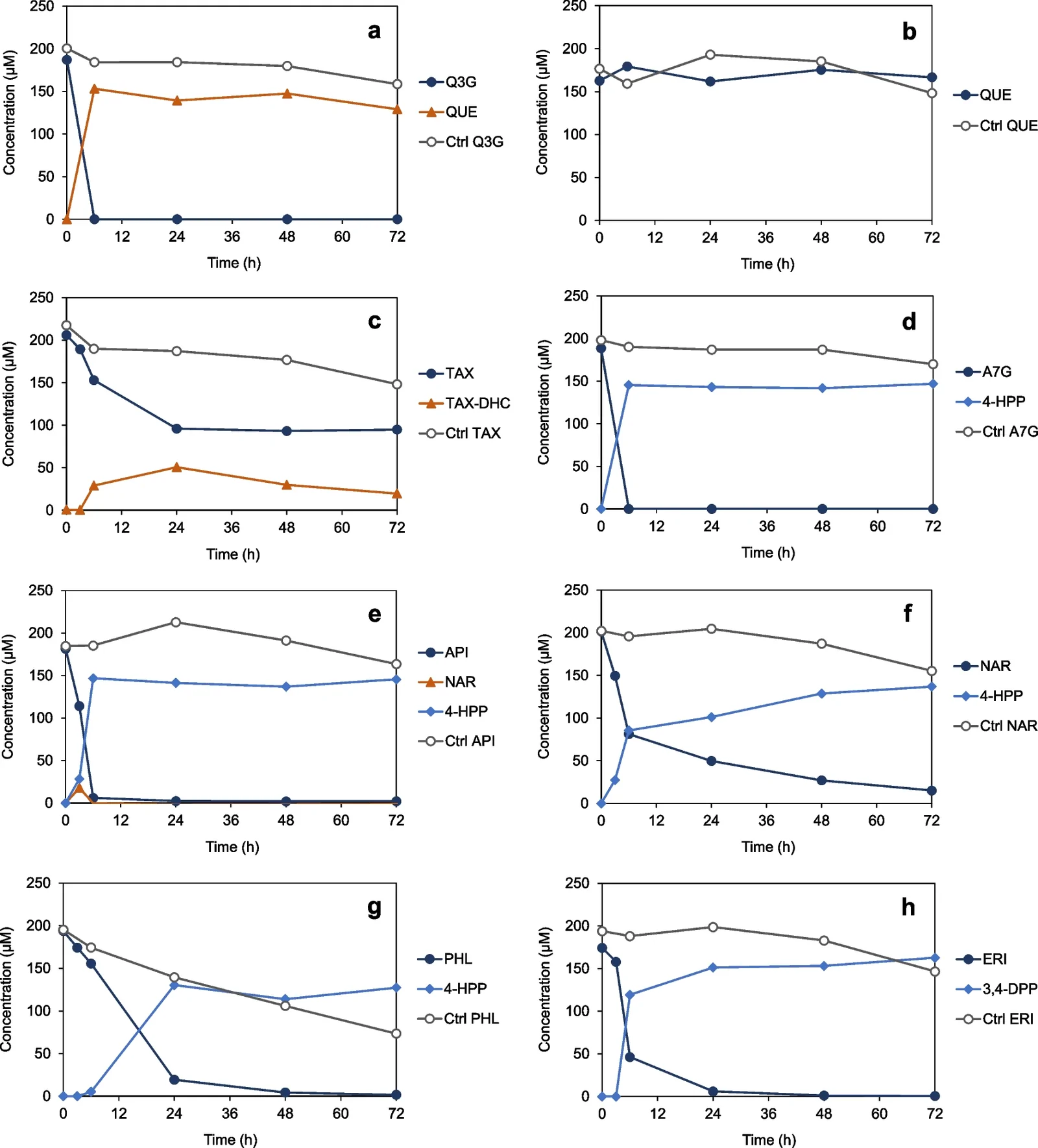
Conversion of flavonoids by *C. butyricum* in RCM_mod_ without glucose: **a** isoquercetin (quercetin-3-*O*-glucoside, Q3G) to quercetin (QUE), **b** directly added QUE was not converted, **c** taxifolin (TAX) to taxifolin dihydrochalcone (TAX-DHC), **d** apigenin-7-*O*-glucoside (A7G) to 3-(4-hydroxyphenyl)propionic acid (4-HPP), **e** apigenin (API) via naringenin (NAR) to 4-HPP, **f** NAR to 4-HPP, **g** phloretin (PHL) to 4-HPP, and **h** eriodictyol (ERI) to 3-(3,4-dihydroxyphenyl)propionic acid (3,4-DPP). As controls (Ctrl), flavonoids were incubated in medium in the absence of *C. butyricum*. Data points are means of triplicates; *SEM* values were < 8%

The glucosylated flavonoids, isoquercetin (quercetin-3-*O*-glucoside) and apigenin-7-*O*-glucoside, were rapidly converted by *C. butyricum* within 6 h of incubation (Fig. 3a and d), even faster than in previous tests without glucose (Fig. 2d and e). Isoquercetin was only deglycosylated. The resulting aglycone, quercetin, was not further degraded, which was confirmed by incubating this flavonol directly with bacterial cultures (Fig. 3b). In contrast, apigenin-7-*O*-glucoside as well as its aglycone apigenin (Fig. 3e) was completely degraded to 4-HPP, the aromatic core of which represents the B-ring of the flavonoid basic structure. The metabolite derived from the flavonoid A-ring, phloroglucinol, was not observed but is known to be rapidly degraded to SCFA by bacteria (Schoefer et al. 2003). In the course of degradation of the flavone apigenin, the flavanone naringenin was detected as intermediate (Fig. 3e). Conversion of both naringenin (Fig. 3f) and phloretin (Fig. 3g) led to formation of 4-HPP. The dihydrochalcone phloretin results from C-ring cleavage of naringenin but is also present as glycoside in plants. Phloretin was cleaved relatively slowly. Corresponding to naringenin, the flavanone eriodictyol was metabolized to 3-(3,4-dihydroxyphenyl)propionic acid (3,4-DPP) (Fig. 3h). Taken together, the half-life of isoquercetin, apigenin-7-*O*-glucoside and apigenin was below 6 h. Half-life of the other flavonoids converted was in some cases higher but overall not comparable due to antibacterial effects or enantiomeric nature of compounds, as discussed below.

The following flavonoids were not metabolized by *C. butyricum* within the entire incubation period of 144 h: quercetin (see above), astilbin (taxifolin-3-*O*-rhamnoside), sakuranetin (7-*O*-methyl-naringenin), isosakuranetin (4′-*O*-methyl-naringenin), kaempferol, dihydrokaempferol, and daidzein (data not shown). Thus, *C. butyricum* was not able to de-rhamnosylate or demethylate flavonoids, which is a prerequisite for subsequent cleavage of the basic flavonoid structure. Moreover, the flavonols and the isoflavone were not attacked. Conversion of flavanonols appeared to depend on the individual structure, as taxifolin was degraded but dihydrokaempferol lacking the hydroxy group at C-3′ was not. Dehydroxylation of the applied flavonoids or resulting phenolic acids was not observed. The results of flavonoid conversion experiments are summarized in Table 1.

**Table 1:** Overview of flavonoids tested for conversion by *C. butyricum* in RCM_mod_ without glucose, formed metabolites, and enzymes probably involved

Flavonoid	Subclass	Metabolite(s)	Enzyme(s)
Isoquercetin	Flavonol	Quercetin	Glu
Quercetin	Flavonol	ND	NA
Kaempferol	Flavonol	ND	NA
Astilbin	Flavanonol	ND	NA
Taxifolin	Flavanonol	TAX-DHC	Fcr
Dihydrokaempferol	Flavanonol	ND	NA
Apigenin-7-*O*-glucoside	Flavone	4-HPP	Glu, FLR, Fcr, Phy
Apigenin	Flavone	Naringenin, 4-HPP	FLR, Fcr, Phy
Eriodictyol	Flavanone	3,4-DPP	Fcr, Phy
Naringenin	Flavanone	4-HPP	Fcr, Phy
Sakuranetin	Flavanone	ND	NA
Isosakuranetin	Flavanone	ND	NA
Phloretin	Dihydrochalcone	4-HPP	Phy
Daidzein	Isoflavone	ND	NA

Chemical structures of flavonoids are shown in Fig. 1. Metabolites: *3,4-DPP *3-(3,4-dihydroxyphenyl)propionic acid, *4-HPP *3-(4-hydroxyphenyl)propionic acid, *TAX-DHC* taxifolin dihydrochalcone

Enzymes: *Fcr* flavanone/flavanonol-cleaving reductase, *FLR* flavone reductase, *Glu* *O*-glucosidase, *Phy* phloretin hydrolase. *NA* not applicable, *ND* not detected

The conversion of the two flavanones, naringenin, and eriodictyol, proceeded initially at high rates but was delayed in further course (Fig. 3f and h). Similarly, the flavanonol taxifolin was only partly converted to a single product within 24 h of incubation (Fig. 3c). The resulting metabolite was subsequently identified as taxifolin dihydrochalcone (structure in Fig. 1) based on comparative analyses with this compound characterized previously (Braune et al. 2019). As flavanones and flavanonols possess (a) chiral center(s), the incomplete conversion indicated their stereospecific cleavage. This was investigated in more detail, and the results are presented in the next chapter.

### Stereospecific conversion of naringenin, eriodictyol and taxifolin by *C. butyricum*

In incubation experiments with *C. butyricum*, racemic specimen of the chiral flavanones, naringenin and eriodictyol, and the flavanonol taxifolin were used. Partial and/or delayed conversion of naringenin, eriodictyol and taxifolin (Fig. 3f, h, and c) indicated each a preference for one of the two enantiomers present. This assumption was corroborated by analyzing samples of conversion experiments by chiral HPLC.

**Fig. 4:**
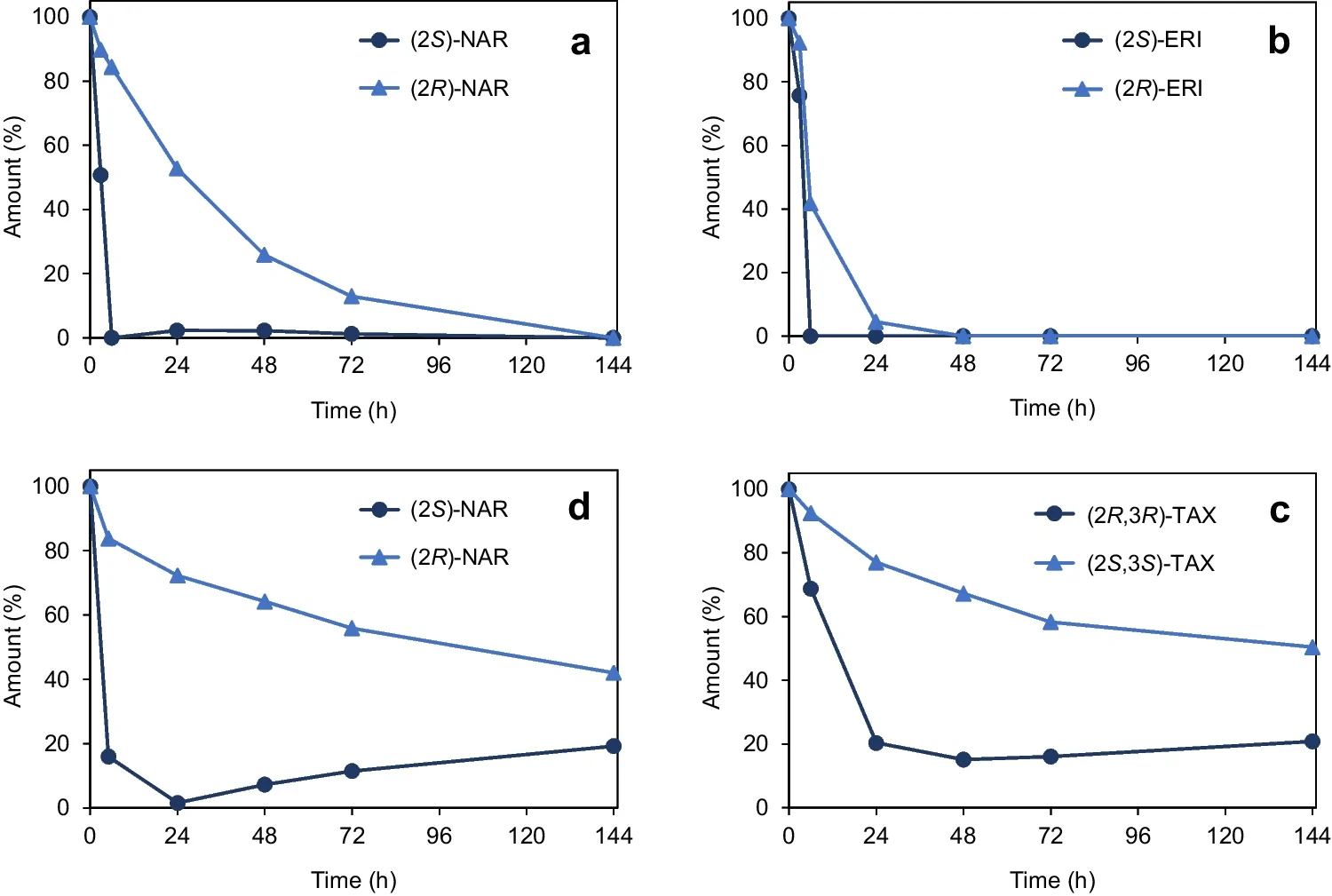
The C-ring cleavage of flavanone and flavanonol members by *C. butyricum* was stereospecific. Chiral HPLC analysis of conversion of racemic **a** naringenin (NAR), **b** eriodictyol (ERI), and **c** taxifolin (TAX) based on individual incubations in RCM_mod_ without glucose shown in Fig. 3. **d** Conversion of NAR based on the incubation in RCM_mod_ with glucose shown in Fig. 2. Percentage values refer to initial concentrations

*C. butyricum* converted only (2*S*)-naringenin (Fig. 4a) and (2*S*)-eriodictyol (Fig. 4b). In parallel, the corresponding (2*R*)-enantiomers disappeared at lower but differing rates, resulting from individual racemization rates of naringenin (*t*_1/2_ = 11.8 h) and eriodictyol (*t*_1/2_ = 60 min) determined previously (Braune et al. 2019). Thus, eriodictyol was converted more rapidly than naringenin due to its much lower half-life and, consequently, increased supply of the accepted (2*S*)-enantiomer.

While naringenin was completely converted in incubation experiments with glucose-free RCM_mod_ within 144 h of incubation (Fig. 2f), approximately only 50% of this flavonoid was degraded within 24 h in medium with glucose, and no further conversion was observed within 144 h (Fig. 2c). In the presence of glucose, *C. butyricum* rapidly grew to high OD_600_ of 7 and reaching the stationary growth phase within 24 h of incubation. Most likely, the associated reduced metabolic activity of bacteria impeded further conversion of naringenin. Chiral analysis revealed that (2*S*)-naringenin was indeed available in incubations with glucose-containing medium at later time points as a potential substrate (Fig. 4d).

Taxifolin was also stereospecifically converted by *C. butyricum*, with preference for the (2*R*,3*R*)-stereoisomer (Fig. 4c). Slowly decreasing concentrations of (2*S*,3*S*)-taxifolin are due to chemical instability of the compound, as can be seen from control incubations in the absence of bacteria (Fig. 3c). Different from flavanones, flavanonols are not subject to spontaneous racemization (Elsinghorst et al. 2011; Kiehlmann and Li 1995). The taxifolin dihydrochalcone formed from taxifolin by *C. butyricum* is also characterized by a chiral carbon (structure in Fig. 1) and, thus, formation of the corresponding enantiomer could be expected. However, an enantiopure standard compound was not available, and resolution of individual enantiomers appears to be difficult by the applied chiral HPLC methods owing to broad peak shape.

**Fig. 5:**
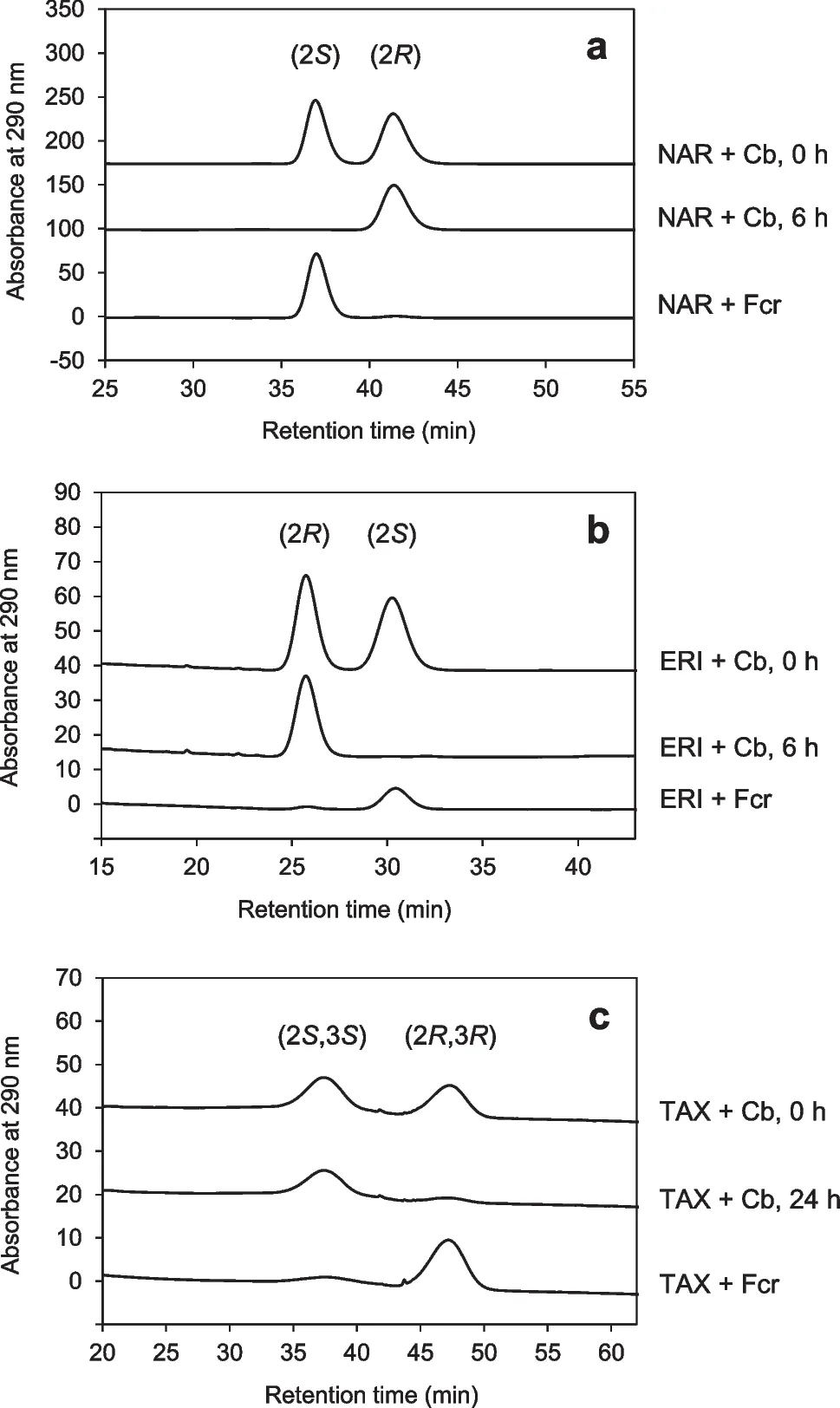
Stereopreference in flavanone and flavanonol degradation by *C. butyricum* differed from that of *E. ramulus*. Chiral HPLC chromatograms of samples collected in the course of conversion of **a** naringenin (NAR), **b** eriodictyol (ERI), and **c** taxifolin (TAX) by *C. butyricum* (Cb) or by the flavanone/flavanonol-cleaving reductase (Fcr) from *E. ramulus*. Samples of conversions by *C. butyricum* are from experiments conducted with glucose-free RCM_mod_

In *Eubacterium ramulus*, the stereospecific heteroring cleavage of flavanones and flavanonols is catalyzed by a NADH-dependent reductase (Fcr) (Braune et al. 2019). This enzyme has been shown to accept (2*R*)-configured eriodictyol and naringenin or (2*S*,3*S*)-configured taxifolin and dihydrokaempferol as substrates. Interestingly, *C. butyricum* converted the other enantiomer of the flavonoids, namely the (2*S*)- and (2*R*,3*R*)-analogues, while dihydrokaempferol was not metabolized. The identity of the respective enantiomers was unequivocally confirmed by re-analyzing samples obtained from previously conducted enzyme assays with Fcr from *E. ramulus* (Braune et al. 2019) in parallel to present samples of *C. butyricum* incubations (Fig. 5).

Naringenin detected as intermediate of apigenin conversion by *C. butyricum* (Fig. 3e) was subsequently identified as the (2*S*)-enantiomer. Its rapid disappearance fits well with the stereopreference in naringenin degradation by *C. butyricum* (Fig. 5a).

### Inhibition of *C. butyricum* growth by phloretin

**Fig. 6:**
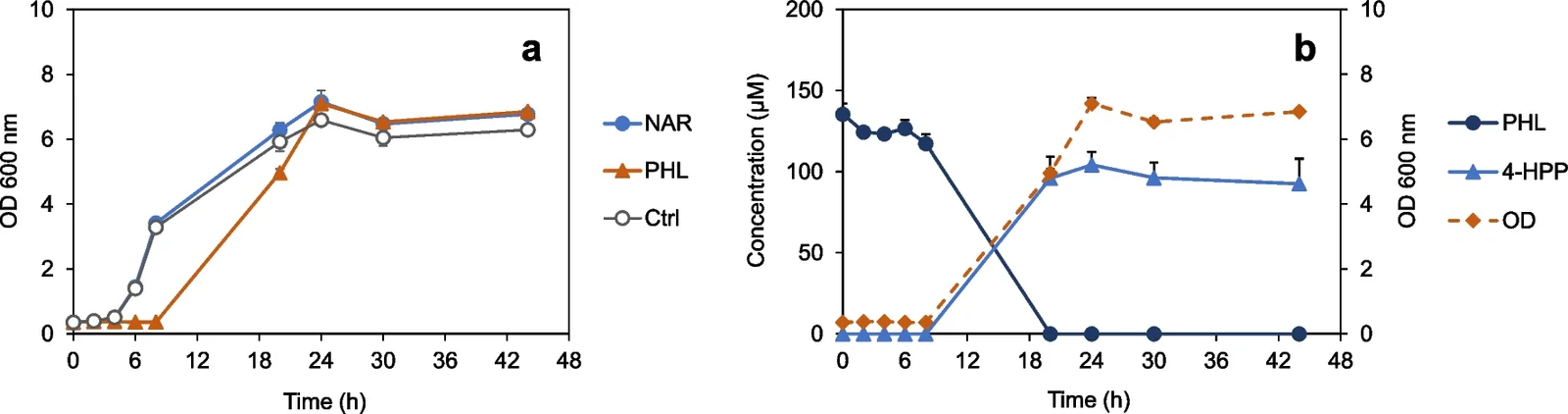
The growth of *C. butyricum* was inhibited by phloretin until it was degraded. **a** Growth of *C. butyricum* in RCM_mod_ with glucose in the presence of naringenin (NAR) or phloretin (PHL). Control (Ctrl) incubation with the solvent DMSO only. **b** Monitoring of PHL conversion to 3-(4-hydroxyphenyl)propionic acid (4-HPP) by *C. butyricum* in parallel to its growth. Broken line refers to the *y* axis on the right. Data points are *means* (± *SEM*) of triplicates. OD, optical density at 600 nm

*C. butyricum* converted apigenin to 4-HPP via naringenin and, likely, phloretin as observed in other human gut bacteria (Braune and Blaut 2016). Naringenin was detected as intermediate (Fig. 3e), and the identical phenolic acid was formed from each of the three flavonoids. Initial conversion rates (0–3 h, *mean* ± *SEM*) of apigenin (21 ± 2 µM/h) and naringenin (17 ± 4 µM/h) were similar (Fig. 3e and f). However, phloretin cleavage proceeded much more slowly (6.5 ± 1.0 µM/h) (Fig. 3g), although only a single enzymatic reaction is required to yield the final product 4-HPP from phloretin. As it is known that phloretin has antibacterial effects (Barreca et al. 2014), the growth of *C. butyricum* in the presence of this dihydrochalcone was monitored and compared to that with naringenin or the DMSO control. In parallel, conversion of the two flavonoids was analyzed. In the control or with naringenin, exponential growth started after 4 h of inoculation (Fig. 6a). The convertible (2*S*)-naringenin enantiomer was completely used up within 4 h of incubation (data not shown), although bacterial density was still low in this time period (Fig. 6a). This was similarly observed (Fig. 4a) with poorly grown cultures in medium lacking glucose. In the presence of phloretin, growth of bacteria was considerably delayed starting not before 8 h of incubation (Fig. 6a) and in parallel to the degradation of this dihydrochalcone to 4-HPP (Fig. 6b).

### Potential flavonoid-converting enzymes encoded in the genome of *C. butyricum*

**Fig. 7:**
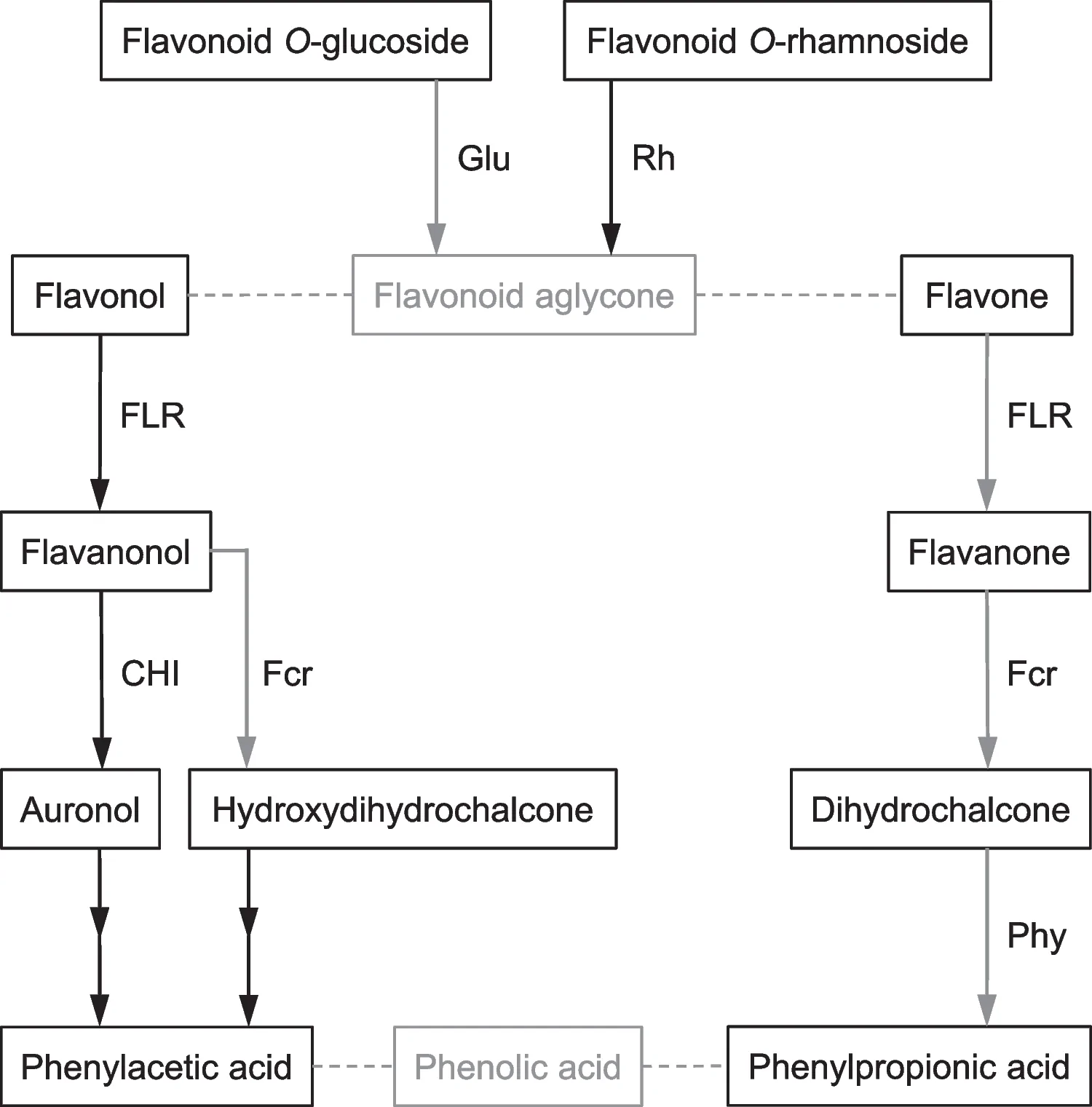
Pathways of flavonoid conversion and involved enzymes in human gut bacteria. Reactions observed in *C. butyricum* are highlighted with blue arrows. CHI, chalcone isomerase; Fcr, flavanone/flavanonol-cleaving reductase; FLR, flavone reductase; Glu, *O*-glucosidase; Phy, phloretin hydrolase; Rh, rhamnosidase

The genome of *C. butyricum* DSM 10702^T^ (GenBank accession number, GCA_014131795) (Xin et al. 2013) was searched for genes that encode potential enzymes catalyzing the flavonoid-converting reactions observed for this bacterium herein (Fig. 7). Protein sequences of functionally characterized bacterial enzymes involved in conversion of flavonoids were used as queries to identify corresponding gene products.

Based on flavonoid-deglycosylating enzymes of gut bacteria reviewed previously (Goris et al. 2021), potential β-glucosidases were found to be encoded in the genome of *C. butyricum* but no rhamnosidases, which correlates with the reactions carried out by this bacterium. Although relative low, most pronounced protein sequence similarities were detected for a predicted glycosyl hydrolase (GH) 3 family β-glucosidase (WP_024041302) of *C. butyricum* with maximal 37% identity to a β-glucosidase from *Bifidobacterium pseudocatenulatum* (KEF28010) acting on isoflavone glycosides (Guadamuro et al. 2017) and a predicted GH 1 family β-glucosidase (WP_033127496) with maximal 36% identity to a β-glucosidase from *Bifidobacterium animalis* ssp. *lactis* (AFS33105) deglycosylating isoflavone and flavonol glycosides (Youn et al. 2012).

A potential flavone reductase (FLR) (QMW92867) was identified according to 70% sequence identity with the FLR from *Clostridium ljungdahlii* DSM 13528^ T^ (ADK16070). Comparison to the FLR from *Flavonifractor plautii* (KGF53654) revealed only 28% identity. This could be explained by the higher degree of relationship of the two clostridia being members of the *Clostridiaceae*. However, the FLRs from *C. ljungdahlii* and *F. plautii* have been shown to reduce the C2–C3 double bond of both flavones and flavonols (Yang et al. 2021), while *C. butyricum* cultures reduced the flavone apigenin to naringenin (Fig. 3e) but did not convert the flavonols quercetin (Fig. 3b) and kaempferol (data not shown).

A potential flavanone/flavanonol-cleaving reductase (Fcr) (QMW92869) is encoded in the *C. butyricum* genome based on a relatively low identity of 32% to the Fcr from *E. ramulus* DSM 16296 (AGS82961). The latter enzyme cleaves the C-ring of flavanones, i.e., naringenin and eriodictyol, and flavanonols, i.e., taxifolin and dihydrokaempferol (Braune et al. 2019). Corresponding reactions were carried out by *C. butyricum* (Fig. 3c, f, and h), except that dihydrokaempferol was not cleaved by this species. Interestingly and as discussed above, the preference for individual flavonoid enantiomers of *C. butyricum* (Fig. 5) differed from that reported for the Fcr from *E. ramulus* (Braune et al. 2019).

A potential phloretin hydrolase (Phy) (QMW92871) was identified based on 57% identity to the Phy from *E. ramulus* DSM 16296 (AAQ12341). According to the characterized *E. ramulus* enzyme (Schoefer et al. 2004), this predicted Phy could be responsible for cleavage of dihydrochalcones as such (e.g., phloretin, Fig. 3g) or as intermediates in the course of flavone/flavanone conversion by *C. butyricum* (Fig. 3d, e, f, and h and Fig. 7).

No potential chalcone isomerase (CHI)-encoding genes could be identified in the genome of *C. butyricum* based on searching with CHI sequences of *E. ramulus* DSM 16296 (AGS82960) or *F. plautii* DSM 4000^ T^ (EHM54434). This further confirms that a CHI is not required for flavone/flavanone cleavage as has been assumed previously (Braune et al. 2019). A C-ring-contracting isomerization of flavanonols to auronols as catalyzed by bacterial CHIs (Braune et al. 2016) was not observed in the present study with *C. butyricum*. Rather, the C-ring of the flavanonol taxifolin was reductively cleaved by an oxidoreductase of *C. butyricum*, possibly the potential Fcr described above (QMW92869), leading to formation of the corresponding hydroxydihydrochalcone.

Remarkably, the three proposed genes encoding FLR, Fcr, and Phy (locus tags: FF104_RS18430, FF104_RS18440, and FF104_RS18450) are consecutively arranged in the genome of *C. butyricum* in an operon-like structure. However, proposed transcription promoter sequences are present upstream of each of the three genes, with transcription start sites at positions − 100, − 45, and − 31, respectively, relative to the translation start of predicted *flr*, *fcr*, and *phy*. Putative Rho-independent transcription terminator sequences are present following the coding regions of *flr* and *phy* in a distance of 35 and 72 nucleotides, respectively. Taken together, this indicates that only the latter two genes, encoding Fcr and Phy, form a transcription unit.

In the genome of *C. ljungdahlii* DSM 13528^ T^, the FLR-encoding gene (CLJU_c30220) and a hypothetical, Phy-encoding open reading frame (CLJU_c30240) are in close proximity, probably also forming a transcription unit (Yang et al. 2021). This potential Phy (ADK16072) shows 53% identity to the Phy from *E. ramulus* DSM 16296 (AAQ12341) and 59% identity to the proposed Phy from *C. butyricum* (QMW92871). Also, a gene encoding a potential Fcr (CLJU_c38590) has been detected but in another region of the *C. ljungdahlii* genome (Yang et al. 2021). The derived Fcr sequence (ADK16884), however, exhibits only low identities of 33% to the characterized Fcr from *E. ramulus* DSM 16296 (AGS82961) and of 36% to the predicted Fcr from *C. butyricum* (QMW92869).

Based on bioinformatic analyses, the potential FLR (QMW92867), Fcr (QMW92869), and Phy (QMW92871) proteins of *C. butyricum* comprise no signal peptide, indicating that these enzymes would not be secreted. Accordingly, the sub-cellular location of FLR and Fcr was predicted as cytoplasmic. For Phy, a transmembrane segment (position 168–189) was detected and, therefore, membrane location suggested. A transmembrane segment (position 181–200) was similarly predicted for Phy from *E. ramulus* DSM 16296 (AAQ12341). Nevertheless, the recombinant *E. ramulus* Phy has been successfully purified from the soluble fraction of *Escherichia coli* (Schoefer et al. 2004). So far, attempts to elucidate the structure of this enzyme have failed (Frank et al. 2014). Based on conserved domains identified in the proposed enzymes of *C. butyricum*, FLR was classified as flavodoxin family protein, Fcr as FAD-dependent oxidoreductase and α/β barrel domain-containing protein, and Phy as member of the DAPG (2,4-diacetylphloroglucinol) hydrolase superfamily.

## Discussion

Gut bacteria play a crucial role in the metabolism of dietary flavonoids, thereby influencing the proposed preventive effects of these plant polyphenols on human health. Knowledge on the involved bacteria and their enzymes is still limited even though these bacterial activities contribute to varying flavonoid-metabolizing phenotypes of human hosts (Cortes-Martin et al. 2020; Morand et al. 2020).

The present studies showed that the common intestinal *C. butyricum* is capable of deglycosylating flavonoid glucosides at different positions but only in the absence of glucose. Most likely, glucose inhibited the flavonoid-deglycosylating enzyme(s). The cleavage of flavonoid *O*-glucosides is catalyzed by β-D-glucoside glucohydrolases (EC 3.2.1.21), belonging to GH family 1, which dominates in bacteria and GH family 3. It is known that these enzymes may substantially be inhibited by their reaction product glucose (Erkanli et al. 2024; Salgado et al. 2018). Thereby, glucose may repress utilization of other carbon sources, such as flavonoid glucosides. In contrast to *C. butyricum*, flavonoid deglycosylation was not found to be inhibited in the presence of glucose in other bacteria, such as *Eubacterium cellulosolvens* or *Catenibacillus decagia,* when incubated in the same medium (Braune and Blaut 2012; Goris and Braune 2024). This indicated that glucose-tolerant β-glucosidases are involved in flavonoid deglycosylation in the latter bacteria.

Moreover, *C. butyricum* was found to be capable of cleaving aglycones of different flavonoid subclasses (Table 1). This also included conversion of the flavanone eriodictyol, as has been reported by Miyake et al. (1997) and had inspired the present study. Thus, *C. butyricum* is the first member of *Clostridiaceae* present in the human gut that has been demonstrated to convert flavonoids. Other human gut bacteria capable of cleaving the C-ring of flavonoids (Braune and Blaut 2016; Goris and Braune 2024) belong to families *Eubacteriaceae*, *Oscillospiraceae*, *Lachnospiraceae* or *Lactobacillaceae* (all phylum *Bacillota*), or to *Eggerthellaceae* (phylum *Actinomycetota*). So far, only a single member of *Clostridiaceae, C. ljungdahlii,* has been shown to degrade flavones and flavonols (Yang et al. 2021). This species was isolated from chicken yard waste (Tanner et al. 1993), indicating its role in flavonoid metabolism in chicken gut. An FLR has been identified in *C. ljungdahlii* following characterization of this enzyme from *F. plautii* (Yang et al. 2021). The FLRs from these two species catalyze the first step in conversion of both flavones and flavonols (Fig. 7), namely reduction of the C2-C3 double bond. In contrast to *F. plautii* and *C. ljungdahlii, C. butyricum* was found to convert the flavone apigenin but not the two tested flavonols, quercetin and kaempferol, suggesting that the involved enzymes differ. However, the FLR from *F. plautii* reduced apigenin to (2*S*)-naringenin (Yang et al. 2021), and this enantiomer equally appeared as intermediate of apigenin conversion by *C. butyricum.* The cleavage of the flavonoid C-ring by *C. butyricum* evidenced a preference for (2*R*)-configured flavanones and (2*S*,3*S*)-configured flavanonols. In *E. ramulus*, this reaction is catalyzed by Fcr, which accepts exactly the other enantiomers (Braune et al. 2019). Flavonoid conversion by *C. butyricum* finally resulted in the formation of phenylpropionic acids, such as 4-HPP and 3,4-DPP. These phenolic metabolites have been discussed as mediators of the health effects attributed to dietary flavonoids, precisely because these compounds are more readily absorbed than their precursors (Carregosa et al. 2022; Obrenovich et al. 2022).

Searching the genome of *C. butyricum* revealed the presence of several genes encoding potential flavonoid-metabolizing enzymes, being in agreement with the observed activities (Table 1). However, the amino acid sequence identity to functionally characterized enzymes ranged widely from 32% (Fcr from *E. ramulus,* Braune et al. 2019) to 70% (FLR from *C. ljungdahlii*, Yang et al. 2021). Potential flavonoid-deglycosylating β-glucosidases, but no rhamnosidases, are encoded, which correlates with the reactions catalyzed by *C. butyricum*. The hypothetical genes encoding an FLR, an Fcr and a Phy in *C. butyricum* were found to be arranged in an operon-like structure suggesting their role in flavonoid degradation pathways (Fig. 7). The combined transcription of corresponding genes has also been discussed for *C. ljungdahlii* (Yang et al. 2021). However, the enzymes predicted in *C. butyricum* need to be further characterized in future studies to provide functional validation.

The strictly anaerobic *C. butyricum* may utilize flavonoids as alternative growth substrates and, as observed for the dihydrochalcone phloretin herein, remove growth-inhibiting flavonoids through degradation. Antibiotic effects of phloretin have been demonstrated in particular for pathogenic bacteria with lowest minimal inhibitory concentrations (MIC) of 8 µM for a Gram-positive species, *Staphylococcus aureus*, and of 125 µM for a Gram-negative one, *Salmonella typhimurium* (Barreca et al. 2014). Less is known about corresponding effects regarding commensal bacteria in the gut. As we observed in a previous study, *F. plautii* did not degrade phloretin at concentrations > 300 µM owing to growth inhibition (Schoefer et al. 2003). The concentration used in the present study with *C. butyricum* of 200 µM inhibited bacterial growth until phloretin was degraded. Mechanistically, impaired growth in the presence of phloretin could be explained by inhibition of the facilitated-diffusion glucose transporter as has been reported for *Bifidobacterium animalis* ssp. *lactis* (Briczinski et al. 2008). Other mechanisms have also been discussed, such as damage to the cell membrane of bacteria leading to leakage (Wang et al. 2020).

The observed flavonoid-metabolizing capabilities of *C. butyricum* might be of considerable relevance not only in the mammalian gut but also in its soil habitat. However, especially the increasing usage of *C. butyricum* strains as probiotics in humans and farm animals has led to growing efforts to further characterize this species and to unravel its health-promoting effects (Stoeva et al. 2021). In this context, the flavonoid-converting activities of *C. butyricum,* as demonstrated herein, should be considered for future applications envisaged for this bacterium.

## Data Availability

All the data supporting the findings of this study are available within the paper.
